# Editorial: Understanding social and psychological effects of social media on contemporary digital consumers

**DOI:** 10.3389/fpsyg.2023.1213731

**Published:** 2023-09-27

**Authors:** Kevin J. Zeng, Cheng-Lu Wang, Morgan X. Yang, Gang Li

**Affiliations:** ^1^Department of Marketing, School of Business, The Hang Seng University of Hong Kong, New Territories, Hong Kong SAR, China; ^2^Marketing Department, Pompea College of Business, University of New Haven, West Haven, CT, United States; ^3^School of Management and Economics, North China University of Water Resources and Electric Power, Zhengzhou, Henan, China

**Keywords:** social media communications, social media marketing, digital consumers, social media influencers, live streaming, community-based behavior

Social media has revolutionized the way individuals communicate by transcending the barriers of time and space (Izogo and Mpinganjira, 2022; Samarah et al., 2022). As of 2022, there were ~4.62 billion social media users worldwide, accounting for 58.4% of the global population. This digital phenomenon has woven itself into numerous aspects of daily life, fostering interconnectedness through platforms specializing in various domains such as food (e.g., Yelp), travel (e.g., TripAdvisor), work (e.g., LinkedIn), and networking (e.g., Facebook and WeChat). With consumers dedicating an increasing amount of time to these diverse platforms, both opportunities and challenges have arisen for businesses and organizations. To stay relevant and effective, they must adapt to the dynamic landscape of social media and harness its potential as an influential communication channel in this interconnected digital age (Gligor and Bozkurt, 2021).

Over the past few years, there has been a significant amount of research conducted on social media communications, including attitude formation and expression (Feng et al., 2021; Yu et al., 2021), electronic word-of-mouth (eWOM; Chan and Yang, 2021; Chan et al., 2022a), platform-based behavior (Chan et al., 2022b; Shukla and Dubey, 2022), customer communications (Yang et al., 2021; Yu et al., 2022; Zeng et al., 2022), and influencer marketing (Dinh and Lee, 2022; Ji et al., 2022; Liao et al., 2023). Despite the immense attention that social media has garnered from both academics and practitioners over the past two decades, our understanding of the social and psychological mechanisms underlying the effects of social media on consumers remains limited due to its fast-paced and ever-changing nature (Wang, 2021). Therefore, the purpose of this Research Topic is to expand and deepen our current understanding of the psychological and social impacts of social media on consumers in an ever-evolving digital landscape, while also challenging and updating the existing literature.

The Research Topic encompasses several areas, one of which is the evolving understanding of social media influencers (SMIs) and their impact on the attitudes, emotions, and behaviors of other social media users (Lee and Eastin, 2021; Ji et al., 2022). SMIs frequently use bragging language to promote luxury brands and their lavish lifestyles (Zhang et al., 2021). Previous research has suggested that humblebragging, a strategy that uses a complaint to disguise self-promotion, is more effective than straightforward bragging. However, the study by Feng et al. found that SMIs' use of humblebragging in luxury brand endorsements negatively affects consumers' brand attitudes. Compared to straightforward bragging, humblebragging triggered malicious envy and reduced trust in the SMI, leading to negative attitudes toward the luxury brand. Furthermore, the negative impact was more pronounced when the SMI lacked expertise or had a high degree of similarity with the consumers. While most studies on SMIs focus on human influencers, Kim et al. explored the effectiveness of brand endorsements by virtual influencers. Virtual influencers have human-like identities and storylines but allow for greater control over content and expression on social media. The results suggested that endorsements from human-like virtual influencers (HVIs) were perceived to be more credible and produced more positive attitudes than endorsements from anime-like virtual influencers (AVIs). However, the superior effect of HVIs disappeared when sponsorship was disclosed.

In addition to influencer marketing, live streaming is a recent trend in social media marketing, and there is still much to understand its social and psychological impact (Deng et al., 2023). Zhang L. et al. conducted a semantic analysis of user attitude formation on various online platforms and discovered that the more product features that are communicated, the lower the perceived price of the product, and the more distant the brand culture, the more difficult it is to form a positive user attitude. However, the platform type mitigated the impact of these factors. Compared to text-based platforms like online review forums, users on live streaming platforms (such as TikTok) demonstrated a more favorable brand attitude toward content with fewer product features, greater cultural distance, and lower perceived price.

Beyond individual-level behavior, three studies have contributed to our understanding of community-based behavior in contemporary social media users. Using longitudinal data from members of an online brand community, Jiang et al. found that brand community participation increased the speed of new product adoption, and the degree of connectedness among community members predicted new product adoption. Furthermore, previous purchase experience positively moderated the relationship between brand community participation and new product adoption. He et al. further explored digital consumers' simultaneous participation in multiple competing brand communities. By using both netnography and survey methods, the authors identified three types of multi-competing brand community engagement (information-oriented, social-oriented, and oppositional) and their key antecedent (product knowledge) and consequence (brand switching). Shifting between online and offline consumer behavior, Xu and Hu highlighted the crucial role of consumer experiences in virtual communities in fostering consumers' perceived value and loyalty, providing evidence of how social media marketing tools can enhance traditional loyalty program-based relationship marketing strategies.

Finally, two articles have provided intriguing insights into how subtle contextual and design factors of social media platforms can shape consumer decisions. Yoon et al. demonstrated that contextual information, such as time and weather, can influence the effectiveness of mobile advertising campaigns on social media. Ads delivered to consumers during their pre-decision stage (vs. decision stage) and under unpleasant (vs. pleasant) weather conditions, increase consumer spending. Zhang N. et al. investigated how a spokes-character's movement on the loading page of a social app influences users' switching intention. The authors found that for hedonic-oriented (utilitarian-oriented) social apps, a high-urgency (low-urgency) spokes-character induced a shorter perceived waiting time, resulting in a lower user switching intention.

In summary, the Research Topic enriches our knowledge of the complex and ever-evolving landscape of social media and its effects on consumers in various ways, including (1) exploring communication style and innovative practices in influencer marketing, (2) investigating user attitude formation on live streaming platforms vs. text-based platforms, (3) examining community-based behavior, and (4) analyzing the impact of contextual and design factors.

## Author contributions

All authors listed have made a substantial, direct, and intellectual contribution to the work and approved it for publication.
